# Extreme Elevation of Alkaline Phosphatase in a Pregnancy Complicated by Gestational Diabetes and Infant with Neonatal Alloimmune Thrombocytopenia

**DOI:** 10.1155/2016/4896487

**Published:** 2016-08-17

**Authors:** Svjetlana Lozo, Amir Atabeygi, Michael Healey

**Affiliations:** Providence St Peter Hospital, 413 Lilly Road NE, Olympia, WA 98506, USA

## Abstract

There have been few case reports of isolated elevation of alkaline phosphatase beyond the normal physiologic amount with subsequent return to baseline after delivery. Here we present a similar case of extreme elevation of alkaline phosphatase in a pregnancy complicated by gestational diabetes and subsequently by neonatal alloimmune thrombocytopenia (NAIT).

## 1. Introduction

Alkaline phosphatase (ALP) is an enzyme found in all tissues throughout the human body. It is concentrated highest in bone, liver, kidney, and intestinal and placental tissue, many of which have their own specific isoenzyme [1]. For this reason, elevated levels of serum alkaline phosphatase are monitored when investigating dysfunction in these tissues. During pregnancy alkaline phosphatase is known to gradually increase, reaching a peak in the third trimester that is around twice its pregestational value [2]. The placental alkaline phosphatase isoenzyme is known to comprise the majority of this increase. We report a case of dramatic elevation in placental alkaline phosphatase associated with right upper quadrant pain during the third trimester of a pregnancy complicated by gestational diabetes. Furthermore, while the delivery itself was an uncomplicated vaginal birth, the infant was found to have diffuse petechiae and purpura secondary to neonatal alloimmune thrombocytopenia.

## 2. Case Report

A 23-year-old woman, gravida 3 para 0, presented for a routine prenatal visit at 34 weeks and 5 days with complaints of constant right upper quadrant pain, headache, and visual disturbance. Serial readings of her blood pressure were all in the normal range and there was minimal proteinuria. Workup revealed an elevation of alkaline phosphatase at 2365 U/L (normal range 35–115 U/L). The patient did not have a history of bone, liver, or renal disease. Past pregnancies were remarkable for a spontaneous abortion and therapeutic abortion requiring dilatation and curettage. Prenatal lab work was unremarkable and the patient was Rh negative. She received RhoGAM during her prenatal care and in the postpartum period. An abdominal ultrasound was performed and failed to show any biliary stones or biliary duct dilatation. To identity the cellular source of the alkaline phosphatase, it was fractionated and showed that 97.8% of the alkaline phosphatase was placental in origin.

During workup the patient was also found to have an elevated glucose tolerance test and was confirmed to have gestational diabetes several days later. The patient was closely monitored for the remainder of the pregnancy and noted to have good glycemic control with dietary modification alone with a hemoglobin A1C of 5.5%. Her alkaline phosphatase was serially monitored and noted to rise to a peak of 4053 U/L (35x upper limit of normal) on the day of delivery. The patient was induced at 40 weeks and 2 days of gestation and had an uneventful vaginal delivery of a vigorous baby boy. Shortly after delivery the infant was noted to have significant bruising and petechiae. A complete blood count was obtained six hours after delivery and revealed significant thrombocytopenia with a platelet count of 20,000. The infant was eventually diagnosed with neonatal alloimmune thrombocytopenia with the parents having an incompatibility in the HPA-1a (PlA1) and HPA-5b (Bra) platelet antigen systems.

**Figure 1:**
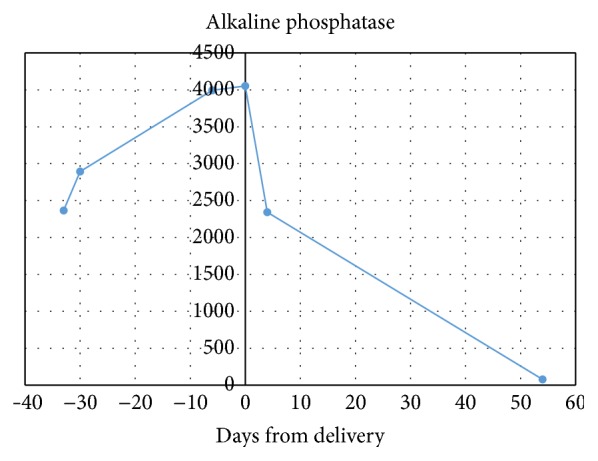
Alkaline phosphate levels trended over course of pregnancy and postpartum period.

Subsequent postpartum monitoring showed the alkaline phosphatase had returned to the reference range after 8 weeks (Figure 1). Placental pathology showed focal mild acute chorioamnionitis and no significant infarction or abnormality.

## 3. Comments

Our literature review found seven cases that demonstrated isolated elevated alkaline phosphatase in the last trimester of pregnancy. Five of the seven cases reported that the placental isoenzyme was the cellular source for this increase.

One of these cases reported a 17-fold increase in alkaline phosphatase that occurred in an uncomplicated pregnancy [3] with 98% of the alkaline phosphatase being placental in origin. In two further cases, placental insult was felt to be the cause of the elevated alkaline phosphatase [2, 4]. In a different case, a 40-year gravida 2 para 1 woman was incidentally found to have elevated alkaline phosphatase at 30 weeks. She was later admitted at 36 weeks for preterm premature rupture of membranes and delivered prematurely via cesarean section for breech presentation. The authors hypothesize in their paper that isolated elevation in placental alkaline phosphatase may be linked to a subsequent preterm delivery [1]. It has been previously postulated that acutely rising alkaline phosphatase could be a biochemical marker for placental injury [5] and/or preterm delivery [6].

The fifth case involved a diet controlled gestational diabetic, similar to our case, though in contrast to our findings and the other documented cases, the authors reported that alkaline phosphatase levels did not return to reference range during the puerperium [7].

In other two cases, elevated alkaline phosphate was found to be due to the bone isoenzyme [8, 9]. One of these cases occurred in a patient with insulin controlled gestational diabetes and hypertension, though the patient was induced and eventually needed emergent cesarean section.

Though it is noteworthy that our patient and two of the above cases were diagnosed with gestational diabetes, it is our belief that this is unlikely the cause of our patient's elevated alkaline phosphatase. Our rationale is that, in all three cases, the patients appeared to have well-controlled blood sugars with either dietary measures or insulin administration. As none of the patients were found to be significantly hyperglycemic, we doubt blood sugar plays a role in our findings.

Our case is the only report in the literature that is associated with a neonatal complication. In neonatal alloimmune thrombocytopenia, maternal antibodies against fetal platelet antigens lead to platelet destruction and subsequent fetal thrombocytopenia. It is possible that these two rare findings of extreme elevation of maternal alkaline phosphatase and NAIT were coincidental. Our literature search did not find any previous reports linking these two findings. One could speculate that this alloimmunization may have led to placental tissue injury and subsequent elevation of alkaline phosphatase. Inconsistent with this hypothesis was the lack of gross placenta abnormality noted on pathologic exam. While it is possible that there is a causal relationship between NAIT and elevated maternal alkaline phosphatase, further studies and reports are necessary to establish any possible link.
